# Single nucleotide polymorphisms in the mitochondrial displacement loop and outcome of esophageal squamous cell carcinoma

**DOI:** 10.1186/1756-9966-29-155

**Published:** 2010-11-26

**Authors:** Ruixing Zhang, Rui Wang, Fengbin Zhang, Chensi Wu, Haiyan Fan, Yan Li, Cuiju Wang, Zhanjun Guo

**Affiliations:** 1 Department of Gastroenterology and Hepatology, The Fourth Hospital of Hebei Medical University. Shijiazhuang, PR China; 2 Department of Thoracic Sugery, The Fourth Hospital of Hebei Medical University, Shijiazhuang, PR China; 3 Department of Molecular Biology, The Fourth Hospital of Hebei Medical University, Shijiazhuang, PR China; 4 Department of Gynecology Ultrasound, The Fourth Hospital of Hebei Medical University, Shijiazhuang, PR China; 5 Hebei Key Lab of Laboratory Animal Science, Hebei Medical University, Shijiazhuang, PR China

## Abstract

**Backgroud:**

Accumulation of single nucleotide polymorphisms (SNPs) in the displacement loop (D-loop) of mitochondrial DNA (mtDNA) has been described for different types of cancers and might be associated with cancer risk and disease outcome. We used a population-based series of esophageal squamous cell carcinoma (ESCC) patients for investigating the prediction power of SNPs in mitochondrial D-loop.

**Methods:**

The D-loop region of mtDNA was sequenced for 60 ESCC patients recorded in the Fourth Hospital of Hebei Medical University between 2003 and 2004. The 5 year survival curve were calculated with the Kaplan-Meier method and compared by the log-rank test at each SNP site, a multivariate survival analysis was also performed with the Cox proportional hazards method.

**Results:**

The SNP sites of nucleotides 16274G/A, 16278C/T and 16399A/G were identified for prediction of post-operational survival by the log-rank test. In an overall multivariate analysis, the 16278 and 16399 alleles were identified as independent predictors of ESCC outcome. The length of survival of patients with the minor allele 16278T genotype was significantly shorter than that of patients with 16278C at the 16278 site (relative risk, 3.001; 95% CI, 1.029 - 8.756; *p *= 0.044). The length of survival of patients with the minor allele 16399G genotype was significantly shorter than that of patients with the more frequent allele 16399A at the 16399 site in ESCC patients (relative risk, 3.483; 95% CI, 1.068 - 11.359; *p *= 0.039).

**Conclusion:**

Genetic polymorphisms in the D-loop are independent prognostic markers for patients with ESCC. Accordingly, the analysis of genetic polymorphisms in the mitochondrial D-loop can help identify patient subgroups at high risk of a poor disease outcome.

## Background

Esophageal cancer is one of the commonest cancers in the population of northern central China with an age-standardized annual incidence rate > 125/100,000 [1]. Cumulative mortality attributed to esophageal cancer is approximately 20% for women and 25% for men [2]. The prognosis of esophageal cancer remains poor, despite improved diagnosis and therapeutic strategies, mostly because of its aggressive nature. The performance status, the TNM stage, and lymph node metastases seem to be the predictive factors of esophageal cancer; some molecular factors, such as p53 mutaion and NF-kappaB expression level, also show predictive power for esophageal cancer outcome [3].

The human mitochondrial genome is 16 kb in length and is a closed-circular duplex molecule that contains 37 genes, including 2 ribosomal RNAs and a complete set of 22 tRNAs [4]. mtDNA is believed to be more susceptible to DNA damage and acquires mutations at a higher rate than nuclear DNA, because of the high levels of reactive oxygen species (ROS), the lack of protective histones, and limited capacity for DNA repair in the mitochondria [5,6].

In cancers patients, sequence changes accumulated extensively in the mitochondrial D-loop region, which is important for regulating both replication and expression of the mitochondrial genome, because it contains the leading-strand origin of replication and the main promoter for transcription [7-10]. Only a few germline single nucleotide polymorphisms (SNPs) in the D-loop have been shown to be prognostic of cancer risk and outcome, but their predictive values have not been fully determined [11-14]. The D-loop contains a length of 1122 bps (nucleotide 16024-16569 and 1-576) refers to mitochondria database (http://www.mitomap.org). In this study, we sequenced a region of about 1 kb in the mitochondrial D-loop from 60 patients with esophageal squamous cell carcinoma (ESCC), the main pathological type of esophageal cancer in China, to assess the relationships between germline SNPs of the D-loop with cancer risk and gemline SNPs with disease outcome in ESCC patients.

## Methods

### Tissue specimens and DNA extraction

Blood samples were collected at the Fourth Hospital of Hebei University from 66 ESCC patients who underwent esophageal cancer resection in the Department of Thoracic Surgery between 2003 and 2004. The patients were selected when they received endoscopy examination and specimen were confirmed as ESCC by pathologist. All the patients comes from the Hebei Province of China a high risk area of ESCC. The tumor-free controls as determined per endoscopy, radiograph, and blood examination, were randomly selected from the same area. Both patients and controls contain 42 males and 24 females with the mean age of 59.78 ± 8.32 in ESCC patients and 60.84 ± 8.77 in controls. Genomic DNA was extracted immediately with a Wizard Genomic DNA extraction kit (Promega, Madison, WI) from blood samples. The study was approved by the Human Tissue Research Committee of the Fourth Hospital of Hebei Medical University. All patients provided written informed consent for the collection of samples and subsequent analysis.

### PCR amplification and sequence analysis

The forward primer 5'-CCCCATGCTTACAAGCAAGT-3' (nucleotide 16190-16209) and reverse primer 5'-GCTTTGAGGAGGTAAGCTAC-3' (nucleotide 602-583) were used for amplification of a 982 bp product from mtDNA D-Loop region as described previously [15]. PCR was performed according to the protocol of PCR Master Mix Kit (Promega, Madison, WI) and purified prior to sequencing. Cycle sequencing was carried out with the Dye Terminator Cycle Sequencing Ready Reaction Kit (Applied Biosystem, Foster City, CA) and the products were then separated on the ABIPRISM Genetic Analyzer 3100 (Applied Biosystem). Polymorphisms were confirmed by repeated analyses from both strands. SNPs were identified directly from blood mitochondria.

### Statistical analysis

The χ^2 ^test was used to analyze dichotomous values, such as the presence or absence of an individual SNP between ESCC patients and healthy controls. The survival curve was calculated using the Kaplan-Meier method, and compared with the log-rank test. Multivariate survival analysis was performed using a Cox proportional hazards model. All of the statistical analysis was done with the SPSS 11.5 software package (SPSS Company, Chicago, IL). A *p *value of < 0.05 was considered statistically significant.

## Results

A total of 66 patients were enrolled in this study. Six of these patients were lost to follow-up. A review was conducted every six months over a five-year period. Those patients lost to follow-up during this time period were as follows: 1 patient in Year 2; 1 patient in Year 3; 3 patients in Year 4; and, 1 patient in Year 5. Sixty patients shared the same performance status (ECOG Score: Zero). Those analyzed included: 12 patients at Stage I; 33 patients at Stage II, and 15 patients at Stage III. Thirty-six patients died during follow-up. None of these patients had received any adjuvant chemotherapy or radiation therapy after ESCC resection. Data for the 5 year follow-up period were analysed with clinical characteristics using the Kaplan-Meier method and were compared by the log-rank test. Sex, age and local lymphatic metastasis were not statistically significant predictors of the length of post-operational survival, but TNM stage was correlated with survival in these patients (Table 1). As expected, patients at different stages had different 5 year survival rates: stage I, 75%, stage II, 36.4% and stage III, 20%. The survival length distribution between any two stages was significantly different (*p *< 0.05) by the log-rank test. These data demonstrated that TNM stage is a good predictor of ESCC outcome.

**Table 1:** Univariate analysis of clinical characteristics associated with post-operational survival in ESCC patients

Characteristics		No. cases	5 years survival rate (%)	*p *value
Gender				0.129
	Male	37	35.10	
	Female	23	47.80	

Age (years)				0.282
	≤ 55	17	23.50	
	> 55	43	46.50	

TNM classification^a^				0.012
	I	12	75	
	II	33	36.40	
	III	15	20	

Lymphatic metastases				0.418
	Yes	12	33.30	
	No	48	41.70	

^a^The survival in each stage was compared as I *versus *II, I *versus *III and II *versus *III

SNPs in reference to GenBank accession AC_000021 were detected in 88 sites of the 982-bp mitochondria D-Loop region from blood samples [see Additional file 1], The sequence chromatograms show a clear single peak at each nucleotide position, indicating that mitochondria in ESCC individuals were homoplasmic. At first, we compared the distribution of germline SNPs at each site between ESCC and control patients to identify any link between an SNP and cancer risk; no association with ESCC cancer risk was detected in any SNP in the D-loop at *p *< 0.05 levels. We assessed the relationships between these SNPs and post-operational survival of these ESCC patients.

**Figure 1:**
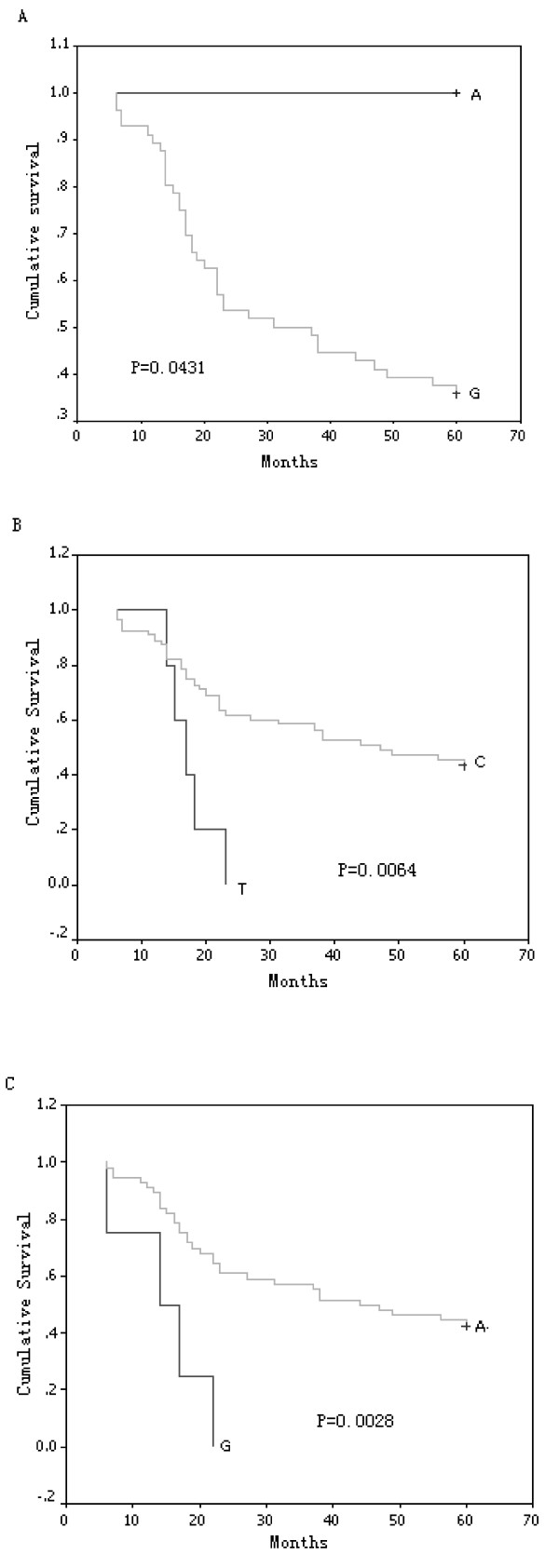
**Survival curve according to the nucleotide at position (A) 16274, (B) 16278 and (C) 16399 in D-loop of ESCC patients**.

The relationship between mtDNA genotype and survival was compared subsequently, the ESCC patients were divided into two groups on the basis of their genotype at each SNP site, the post-operational survival curve was plotted using the Kaplan-Meier method for all ESCC patients at these sites. A dramatic difference in survival rate appeared at 16274, 16278 (refers to rs41458645 in NCBI SNP database, http://www.ncbi.nlm.nih.gov/snp/) and 16399 alleles by the log-rank test (Figure 1). The 3 SNPs were previously identified in mitochondria database (http://www.mitomap.org). The frequent allele 16274G, and the rare alleles 16278T and 16399G were associated with a shorter period of survival, with *p *= 0.0431, 0.0064 and 0.0028, respectively (Figure 1A, B and 1C). We performed multivariate analysis with Cox proportional hazards model including the factors of three SNPs and TNM stage. We coded G as 0 and A as 1 at 16274 position, C as 0 and T as 1 at 16278, A as 0 and G as 1 at 16399. We also coded I of TNM stage as 0, II as 1 and III as 2. As shown in Table 2, the 16278 and 16399 alleles were identified as independent predictors for ESCC outcome. The length of survival for patients with the rare allele 16278T genotype was significantly shorter than that for patients with the frequent allele 16278C (relative risk, 3.001; 95% CI, 1.029 - 8.756; *p *= 0.044) at the 16278 site. The same was seen for the rare allele 16399G genotype when compared with matched alleles 16399A at the 16399 site in ESCC patients (relative risk, 3.483; 95% CI, 1.068 - 11.359; *p *= 0.039) (Table 2). These data demonstrated the strong prediction power of 16278C/T and 16399A/G on outcome for ESCC patients.

**Table 2:** Multivariate analysis of prognostic factors associated with post-operational survival in ESCC patients with Cox proportional hazards model

Factors	Relative risk	95% C.I.	*p *value
Stage of tumor	1.328	0.955-1.848	0.092

16274(G/A)	0	0	0.975

16278(C/T)	3.001	1.029-8.756	0.044

16399((A/G)	3.483	1.068-11.359	0.039

## Discussion

Selected SNPs in the D-loop region have been examined for the ability to predict cancer risk in other types of tumour [11-14]. The present study has extended those analyses to determine the cancer risk and the post-operational survival-associated germline SNPs in a continuous sequence of mtDNA between nucleotides 16190 and 583 in ESCC patients. Three SNPs, 16274G/A, 16278C/T and 16399A/G, were identified for their association with post-operational survival at statistically significant levels by the log-rank test. Multivariate survival analysis identified 16278C/T and 16399A/G to be independent prediction markers for ESCC outcome.

We suggest for the first time that SNPs in the D-loop is a prognostic factor in ESCC patients. The relative risk (RR) of death in patients was significantly higher (16278C *versus *16278T, RR, 3.001; 95% CI, 1.029 - 8.756; *p *= 0.044. 16399A *versus *16399G, RR, 3.483; 95% CI, 1.068 - 11.359; *p *= 0.039). Nucleotides 16278 and 16399 are located in hypervariable segment 1 (HV1), which is associated with high rates of mutation [16], but the functional significance of these SNPs in HV1 is not known. Minor alleles of 16278T and 16399G are associated with dramatically shorter period of postoperative survival; the survival curve decreased rapidly in patients carrying these alleles (Figure 1). We compared the distribution frequency of these two SNPs between ESCC patients and normal controls; among 60 age-matched controls, only one carried the 16278T allele and none carried the 16399G allele. These two minor alleles appear to be correlated with ESCC risk, but a much larger sample size will be needed to obtain a statistically significant difference of distribution frequency for these alleles between ESCC patients and controls. It is very interesting if a nucleotide substitution can render an individual susceptible to a tumour but subsequently modulates prognosis of that tumour. The 5 year survival rate displays a trend for distinction on the basis of sex (35.1% male *versus *47.8% female), age (46.5% older than 55 *versus *23.5% younger than 55) and lymphatic metastasis (33.3% yes *versus *41.7% no). The prediction value of these factors need to be investigated by further study.

The D-loop region of mtDNA is important for regulation of mitochondrial genome replication and expression. SNPs in this region might affect mtDNA replication and lead to alteration of the electron transport chain, which is responsible for the release of highly reactive oxygen species (ROS) and could contribute to nuclear genome damage as well as cancer initiation and promotion [17-19]. These three SNPs may altered transcription of mitochondrial genome, and that the production of ROS is enhanced when the mitochondrial transcription is altered [20], these ROS-mediated mechanism may accelerate the tumor development.

In conclusion, SNPs in the D-loop were found to be independent prognostic markers for ESCC outcome. The analysis of genetic polymorphisms in the D-loop might help to identify patient subgroups at high risk for a disease outcome, thereby helping to refine therapeutic decisions in ESCC cancers.

## Competing interests

The authors declare that they have no competing interests.

## Authors' contributions

RZ and RW contributed to experimental design, data acquisition and analyses. FZ, CW and FHY contributed to experimental design, specimen collection, and data acquisition. YL and CW participated in data analyses. ZG contributed to conception, experimental design, data acquisition, analyses, and interpretation, and manuscript preparation. All authors have read and approved the final manuscript.

## Supplementary Material

Additional file 1 **Distribution of 88 SNPs in 66 ESCC patients and controls**. The data provided represent all the SNPs identified in the ESCC patients and controls. Click here for file
